# Hypercapnic acidosis induces mitochondrial dysfunction and impairs the ability of mesenchymal stem cells to promote distal lung epithelial repair

**DOI:** 10.1096/fj.201802056R

**Published:** 2019-01-16

**Authors:** Nicola Fergie, Naomi Todd, Lana McClements, Danny McAuley, Cecilia O’Kane, Anna Krasnodembskaya

**Affiliations:** Centre for Experimental Medicine, School of Medicine, Dentistry, and Biomedical Sciences, Queen’s University of Belfast, Belfast, United Kingdom

**Keywords:** ARDS, hypercapnic acidosis, mitochondrial transfer, wound healing

## Abstract

Acute respiratory distress syndrome (ARDS) is a devastating disorder characterized by diffuse inflammation and edema formation. The main management strategy, low tidal volume ventilation, can be associated with the development of hypercapnic acidosis (HCA). Mesenchymal stem cells (MSCs) are a promising therapeutic candidate currently in early-phase clinical trials. The effects of HCA on the alveolar epithelium and capillary endothelium are not well established. The therapeutic efficacy of MSCs has never been reported in HCA. In the present study, we evaluated the effects of HCA on inflammatory response and reparative potential of the primary human small airway epithelial and lung microvasculature endothelial cells as well as on the capacity of bone marrow−derived MSCs to promote wound healing *in vitro*. We demonstrate that HCA attenuates the inflammatory response and reparative potential of primary human small airway epithelium and capillary endothelium and induces mitochondrial dysfunction. It was found that MSCs promote lung epithelial wound repair *via* the transfer of functional mitochondria; however, this proreparative effect of MSCs was lost in the setting of HCA. Therefore, HCA may adversely impact recovery from ARDS at the cellular level, whereas MSCs may not be therapeutically beneficial in patients with ARDS who develop HCA.—Fergie, N., Todd, N., McClements, L., McAuley, D., O’Kane, C., Krasnodembskaya, A. Hypercapnic acidosis induces mitochondrial dysfunction and impairs the ability of mesenchymal stem cells to promote distal lung epithelial repair.

Acute respiratory distress syndrome (ARDS) is an acute inflammatory disorder in which the integrity of the alveolar epithelial−capillary endothelial barrier is compromised and protein-rich alveolar edema accumulates. Low tidal volume ventilation is the mainstay of treatment, but it can be associated with the development of hypercapnic acidosis (HCA) (1, 2). Although secondary analysis of clinical data suggested that HCA may reduce mortality in ARDS (3), data from preclinical models are controversial.

The pulmonary capillary endothelium and alveolar epithelium contribute to inflammation in ARDS by promoting alveolar neutrophil recruitment. In addition, disruption to the integrity of the endothelial-epithelial barrier contributes to the accumulation of pulmonary edema. The response of these endothelial and epithelial cells to HCA may therefore have profound implications for the resolution of ARDS.

To date, only 2 studies have reported the effects of HCA on endothelia. These studies have highlighted conflicting results; whereas Takeshita *et al.* (4) reported attenuation of inflammation by HCA, Liu *et al.* (5) demonstrated that HCA enhances inflammatory responses. It is noteworthy that Takeshita *et al.* modeled the endothelium using macrovascular human pulmonary artery endothelial cells, whereas Liu *et al.* utilized human pulmonary microvascular endothelial cells (HPMECs). Significant heterogeneity exists between macrovascular and microvascular cells with regard to protein expression profiles and barrier function (6–10). The use of human pulmonary artery endothelial cells to study the pulmonary capillary endothelium may therefore limit the translational value of the results obtained. Furthermore, although HPMECs are the most relevant cell type in the context of ARDS, in the study by Liu *et al.*, the *in vitro* results were corroborated by data in a rabbit model of LPS-induced lung injury in which endothelial-neutrophil responses were significantly increased during hypercapnia (5). These data contradict previous *in vivo* findings in the models of sepsis- and paraquat-induced lung injury in rats, demonstrating an immunosuppressive effect of HCA (11, 12).

Although several studies reported that HCA attenuates the contribution of the alveolar epithelium to inflammation (13, 14)—an effect that would be beneficial in ARDS—other *in vitro* research indicates that it may also attenuate wound closure (15) and alveolar fluid clearance (16–20), suggesting impaired potential for alveolar re-epithelialization and the resolution of pulmonary edema. However, much of this work was performed in the adenocarcinomic human alveolar basal epithelial cell line A549. Although generally considered representative of the alveolar epithelium, concerns exist regarding the consistency of the A549 phenotype compared with that of primary human alveolar epithelial cells (21–26). Results obtained in A549 cells should therefore be interpreted with caution until confirmed in primary cells.

Although no pharmacological therapy has been successful in treating ARDS (27), mesenchymal stem cells (MSCs) show promising therapeutic potential against inflammation and pulmonary edema in preclinical models (28–31). These effects may be mediated by the secretion of paracrine mediators (32–34) or *via* transfer of mitochondria to injured cells (35, 36). MSCs have entered early-phase clinical trials, which to date attest to their safety in ARDS (37–39). However, although known to respond to their local environment, the effects of HCA on MSC biology and therapeutic potential have never been reported.

The aims of the present work were as follows: *1*) determine the effect of HCA on the inflammatory and reparative responses of the human capillary endothelium and alveolar epithelium, *2*) determine the effect of HCA on the biologic properties and therapeutic capacity of MSCs, and *3*) elucidate the mechanisms of these effects. Some of the results of these studies were previously reported in the form of an abstract (40).

## MATERIALS AND METHODS

### Cell culture

HPMECs and human small airway epithelial cells (SAECs) (both from PromoCell, Heidelberg, Germany) were maintained in endothelial cell growth medium MV and SAEC growth medium (PromoCell), respectively. Each medium was supplemented with 50 µg/ml penicillin-streptomycin (PS) (Thermo Fisher Scientific, Waltham, MA). Both cell types were used to passage 7. Human bone marrow−derived MSCs met the criteria of the International Society for Cellular Therapy (41) and were obtained from the Institute for Regenerative Medicine at Texas A&M Health Science Center (Temple, TX, USA). MSCs were maintained in minimum essential medium with alpha modifications (α-MEM) (Thermo Fisher Scientific) supplemented with 16.5% heat-inactivated fetal bovine serum (FBS), 50 µg/ml PS, and 4 mM l-glutamine (LG) (Thermo Fisher Scientific) and were used to passage 5.

### Induction of HCA

Conditions of normocapnia and HCA were achieved using Panasonic (Osaka, Japan) MCO-230AIC CO_2_ incubators, with CO_2_ set to 5 and 15%, respectively. Media used for experimentation were pre-equilibrated in the absence of cells in T25 cell culture flasks under these conditions overnight. pH and partial pressure of CO_2_ (pCO_2_) were analyzed using a cobas b 221 blood gas analyzer (Roche, Basel, Switzerland).

### Inflammatory stimulation of cells

Unless otherwise stated, HPMECs or SAECs were seeded at a density of 1 × 10^4^ cells/cm^2^ and allowed to reach 80% confluence in 5% CO_2_ prior to use for experimentation. Media were then removed, and the cells were washed with Dulbecco’s phosphate-buffered saline (DPBS). For experimentation, cells were stimulated with a cytomix of proinflammatory cytokines. This cytomix was prepared separately in pre-equilibrated cell culture media for cells to be exposed to normocapnia and for cells to be exposed to HCA. The cytomix contained TNF-α (Peprotech, Rocky Hill, NJ, USA), IL-1β (Peprotech), and IFN-γ (R&D Systems, Minneapolis, MN, USA), each at a concentration of 50 ng/ml. Stimulated cells were subsequently cultured in either 5% CO_2_ (normocapnia; pCO_2_ 3.4 ± 0.1 kPa and pH 7.3) or 15% CO_2_ (HCA; pCO_2_ 9.0 ± 0.2 kPa and pH 7.0) for the duration of each experiment. Unless otherwise stated, HPMECs and SAECs were exposed to 5 or 15% CO_2_ with or without cytomix for 72 and 24 h, respectively, before analyses were performed. In contrast, MSCs were seeded in the presence or absence of cytomix and cultured immediately in 5 or 15% CO_2_ at the time of seeding. For experiments in which MSCs were the sole cell type used, cells were seeded at a density of 2 × 10^4^ cells/cm^2^ and analyses were performed after 24-h culture in 5 or 15% CO_2_ with or without cytomix.

### Coculture experiments

For coculture experiments, MSCs were seeded on Transwell inserts (Greiner Bio-One, Kremsmünster, Austria) at a ratio of 1:5 MSC:SAECs. SAECs had been seeded at a density of 1 × 10^4^ cells/cm^2^ and allowed to reach 80% confluence in 5% CO_2_. Medium was then aspirated, and the cells were washed with DPBS before the addition of MSCs. Cytomix stimulation was applied to both the upper and lower chambers at the time of MSC seeding. The coculture was then immediately incubated in 5 or 15% CO_2_ for 24 h before analyses were performed.

### Quantification of cytokines and growth factors by ELISA

Commercially available DuoSet Sandwich ELISAs (R&D Systems) were used to measure human C-X-C motif chemokine ligand (CXCL)8, CXCL5, angiopoietin-1 (Ang-1), keratinocyte growth factor, IL-1 receptor antagonist (IL-1ra), and IL-10 concentrations in cell-free supernatants. All assays were performed following the manufacturer’s standard instructions for DuoSet ELISAs. Working concentrations of the antibodies provided with each kit were analyte and lot specific.

### Evaluation of endothelial adhesion molecule expression by HPMECs

Flow cytometry was used to assess the effects of HCA on HPMEC expression of the adhesion molecules endothelial-selectin (E-selectin), intercellular adhesion molecule (ICAM)-1, and VCAM-1.

After 72 h incubation in the presence or absence of 50 ng/ml cytomix, HPMEC monolayers were washed with 1 ml 1× PBS, and the cells were gently scraped, transferred into 5-ml polystyrene flow tubes (Sarstedt; Thermo Fisher Scientific), and centrifuged for 5 min at 800 rpm and 4°C. The supernatant was aspirated off, and the pellet resuspended in 200 µl 1× PBS. To prevent non-specific binding of antibodies, 20 µl human Fc Receptor binding inhibitor (Thermo Fisher Scientific) was added and incubated with the cells on ice for 20 min. Appropriate antibodies directed against the proteins of interest (**Table 1**) were added to the experimental groups and incubated on ice for 30 min in the dark. Unstained controls, isotype controls for each fluorophore used, and single-stain controls for each fluorophore were also included. Samples were immediately processed using a FACSCanto II flow cytometer and FACSDiva software (BD Biosciences, San Jose, CA, USA). If necessary, compensation was set up using FACSDiva between 2 fluorophores based on overlap in the single-stain controls. All data were analyzed using FlowJo v.10 software (FlowJo; BD Biosciences, San Jose, CA, USA).

**TABLE 1:** Antibodies used to assess HPMEC adhesion molecule expression by flow cytometry

Protein of interest	Fluorophore	Manufacturer	Dilution
Anti-human CD62E (E-selectin)	APC	BioLegend	1:100
Anti-human CD54 (ICAM-1)	FITC	BioLegend	1:100
Anti-human CD106 (VCAM-1)	PE	BioLegend	1:100

APC, Allophycocyanin; CD, cluster of differentiation; PE, Phycoerythrin.

### *In vitro* wound scratch assay

An *in vitro* wound scratch assay was used to assess the effects of HCA on epithelial and endothelial wound repair. Horizontal lines were created across the undersurface of the wells of 24-well plates prior to cell seeding. HPMECs or SAECs were seeded on these plates at a density of 1 × 10^4^ cells/cm^2^ and cultured until monolayer formation occurred. At this point, a single vertical scratch wound was made from the top to the bottom of each well, running through the horizontal line, using a P1000 pipette tip (Sarstedt, Nümbrecht, Germany). The edge of a ruler was used to guide a straight line. Cells were washed twice with DPBS to remove cell debris, and 500 µl 1% supplemented medium (see negative control in **Table 2**) was added to each of the wells. The wound sites were imaged at ×10 magnification using the Axiovert 25 inverted light microscope (Carl Zeiss, Oberkochen, Germany) and AxioVision Release 4.8 software (Carl Zeiss). Two images were taken from each well; 1 was taken just above the horizontal line and 1 just below it to allow for reimaging of the same area of each wound later in the experiment. To limit bias when calculating wound closure, each of the conditions was blinded by an independent researcher unrelated to the project prior to its addition to the cells. Wounded cells, regardless of cell type, were incubated under appropriate experimental conditions for 24 h at 37°C in either 5 or 15% CO_2_.

**TABLE 2:** Conditions added to wounded HPMECs and SAECs

Conditions	HPMEC (Endothelial cell growth medium MV)	SAEC medium
Vehicle	10% supplemented	10% supplemented
Cytomix	50 ng/ml cytomix (TNF-α, IL-1β, IFN-γ) in vehicle	50 ng/ml cytomix (TNF-α, IFN-γ) in vehicle
Negative control	1% supplemented	1% supplemented
Positive control	100% supplemented	100% supplemented

Percentage supplement refers to the percentage of the supplement added to the

standard culture medium for each cell type.

Wounds were reimaged as before in the same areas as the baseline wounds following 24 h incubation. ImageJ v.1.48 (National Institutes of Health, Bethesda, MA, USA) was used to measure the area of the wound site at baseline and again at 24 h. The percentage wound closure at 24 h was calculated from these values. Only at this stage were the conditions unblinded to permit interpretation of the results.

### Determination of cell viability by lactate dehydrogenase assay

The commercially available Cytotoxicity Detection Kit (Roche), which is based on the activity of lactate dehydrogenase (LDH) in cell-free supernatants, was used to assess the impact of HCA on HPMEC, SAEC, and MSC cell death in both inflammatory and noninflammatory environments.

At 24, 48, or 72 h post-stimulation of cells with cytomix, cell supernatants were collected and centrifuged to remove cell debris. A positive control was included in which cells were lysed with a final concentration of 2% Triton X-100 (MilliporeSigma, Burlington, MA, USA) 10 min before the collection of supernatants. Fifty-microliter samples were plated in triplicate into a 96-well plate (Nunc; Thermo Fisher Scientific). Optical densities were measured at 405 nm using a Fluostar Omega (BMG Labtech, Ortenberg, Germany) microplate reader. Data were analyzed using Mars data analysis software (BMG Labtech). Results are presented relative to the positive control.

### NF-κB activation

#### Stimulation of cells

MSCs were seeded on 6-well plates at a density of 2 × 10^5^ cells per well and allowed to adhere overnight in α-MEM_16.5% FBS + PS + LG_. HPMECs or SAECs were seeded on 6-well plates at a density of 1 × 10^5^ cells per well and cultured in their respective cell culture medium until monolayer formation was achieved. On the day of experimentation, the cells were washed with 1 ml prewarmed DPBS, which was immediately aspirated off. Two milliliters α-MEM_1% FBS + PS + LG_, which had been pre-equilibrated in 5 or 15% CO_2_ overnight, was added to the MSCs, and the cells were left to rest for 2 h in 5 or 15% CO_2_ at 37°C because of the sensitivity of NF-κB activation. For HPMECs or SAECs, their respective cell culture medium was used. After incubation, 100 µl medium was removed from each well. Cytomix was prepared to a concentration of 1 µg/ml in pre-equilibrated α-MEM_1% FBS + PS + LG_ for MSCs or cell cultured medium for HPMECs and SAEC. A total of 100 µl was added to the 1.9 ml medium already in the appropriate wells, bringing the final concentration to 50 ng/ml. For un-stimulated cells and for the 0-min control, 100 µl of only pre-equilibrated medium was added instead of cytomix. The plates were incubated at 37°C and either 5 or 15% CO_2_ for 0, 30, or 60 min. At the end of each incubation period, the medium was aspirated from the wells, and the cells were scraped from the surface in 1 ml ice-cold PBS. Replicate cell suspensions of each condition were pooled and kept on ice in preparation for nuclear extraction.

#### Preparation of nuclear extracts

Nuclear extracts were prepared from chilled cell suspensions using the NE-PER Nuclear and Cytoplasmic Extraction Reagents Kit (Thermo Fisher Scientific), following the manufacturer’s instructions. Briefly, the cells were pelleted by centrifugation at 800 *g* for 3 min at 4°C, and the supernatant was discarded. The cell pellet was resuspended in 200 µl Cytoplasmic Extraction Reagent I containing Halt protease inhibitor cocktail (EDTA-free) (Thermo Fisher Scientific), vortexed, and incubated for 10 min on ice before the addition of 11 µl Cytoplasmic Extraction Reagent II. Samples were vortexed at the highest setting for 5 s, incubated on ice for 1 min, and vortexed again before centrifugation at 16,000 *g* for 5 min at 4°C. Supernatants containing cytoplasmic extracts were collected into ice-cold Eppendorf tubes and kept on ice until the end of the extraction protocol.

The insoluble pellet was resuspended in 100 µl ice-cold Nuclear Extraction Reagent containing Halt protease inhibitor cocktail (EDTA-free) and vortexed at the highest setting for 15 s. Samples were incubated on ice for 40 min, with the vortexing step repeated every 10 min until the time was up. Supernatants containing the nuclear extracts were collected into fresh, ice-cold Eppendorf tubes immediately following centrifugation of the samples at 16,000 *g* for 10 min at 4°C. All extracts were stored at −80°C for future use.

#### Protein quantification

Protein concentrations in nuclear extracts were quantified using the Coomassie (Bradford) Protein Assay Kit (Thermo Fisher Scientific). Bovine serum albumin (BSA) standards ranging in concentration from 0 to 2000 µg/ml were prepared following the manufacturer’s instructions. A total of 5 µl of each standard or sample was pipetted in duplicate into the wells of a 96-well plate followed by 250 µl Coomassie reagent. Well contents were mixed on a plate shaker for 30 s, and the plate was incubated at room temperature for 5 min, protected from light. Absorbance was measured at 595 nm using a Fluostar Omega microplate reader, and the concentration of protein in each unknown sample was extrapolated from a second polynomial curve generated using Mars data analysis software.

#### TransAM NF-κB p65 assay

The TransAM NF-κB p65 Assay Kit (Active Motif, Carlsbad, CA, USA) is a DNA-binding ELISA that facilitates the detection of active NF-κB p65 subunits in nuclear extracts. In this assay, active p65 subunits bind to their consensus oligonucleotides, which have been immobilized on a 96-well plate supplied with the kit. Bound subunits can be detected thereafter in a principle similar to that of an ELISA. This assay was used, following the manufacturer’s instructions, to detect active p65 subunits in the nuclear extracts of HPMECs, SAECs, or MSCs cultured in 5 or 15% CO_2_.

Nuclear extracts were diluted in complete lysis buffer to a protein concentration of 150 µg/ml (for MSCs) or 100 µg/ml (for HPMECs or SAECs). Jurkat extract (positive control supplied with the kit) was also diluted to a protein concentration of 125 or 100 µg/ml in complete lysis buffer. A total of 30 µl complete lysis buffer was added to the wells of the 96-well plate supplied followed by 20 µl Jurkat extract or unknown sample (containing 3 µg protein for MSCs or 2 µg protein for HPMECs and SAECs). A total of 20 µl complete lysis buffer was included as a blank control, and the plate was incubated at room temperature for 1 h with mild agitation from a plate rocker. The plate was washed 3 times using the wash buffer supplied with the kit and gently blotted against paper towels to dry. Primary antibody (rabbit anti−NF-κB p65) was diluted 1:1000, and 100 µl was added per well. The plate was incubated at room temperature for 1 h in the absence of agitation from a plate rocker. The wash step was repeated and 100 µl Horseradish Peroxidase-conjugated anti-rabbit secondary antibody (diluted 1:1000) was added per well. The plate was incubated again for 1 h at room temperature without agitation. The wash step was repeated 4 times. A total of 100 µl developing solution was added per well and incubated at room temperature protected from light until the color of the sample wells had turned medium-to-dark blue. A total of 100 µl stop solution was added to stop the reaction, and the optical density was measured within 5 min at 450 nm using a Fluostar Omega plate reader.

### Assessment of mitochondrial function

#### Measurement of mitochondrial membrane potential using 5,5′,6,6′-tetrachloro-1,1′,3,3′-tetraethylbenzimidazolylcarbocyanine iodide dye

5,5′,6,6′-tetrachloro-1,1′,3,3′-tetraethylbenzimidazolylcarbocyanine iodide (JC-1) is a lipophilic, cationic dye that accumulates within mitochondria in a manner dependent on mitochondrial membrane potential. When mitochondrial membrane potential is low, JC-1 accumulates within mitochondria in low concentrations and persists in a monomeric form, fluorescing green. At higher mitochondrial membrane potential, JC-1 accumulation within the mitochondria is increased. Red fluorescing aggregates begin to form. A higher red/green fluorescence ratio is therefore proportional to mitochondrial membrane potential. JC-1 was used to assess the impact of HCA on mitochondrial membrane potential in HPMECs, SAECs, and MSCs under both inflammatory and noninflammatory conditions.

After 24 h of cytomix stimulation and culture in 5 or 15% CO_2_, cells were stained with JC-1 (Thermo Fisher Scientific) at a concentration of 0.5 µg/ml for 45 min (SAECs) or 1 h (HPMECs and MSCs). A control was included in which 1 µM carbonyl cyanide p-trifluoromethoxyphenylhydrazone (FCCP) (MilliporeSigma) was simultaneously added to the cells at the time of JC-1 staining to induce mitochondrial depolarization (collapse of mitochondrial membrane potential). Following staining, wells were washed 3 times using 1× PBS. A total of 100 µl 1× PBS was added per well. Live cells were immediately imaged at ×20 magnification using the EVOS FL Auto epifluorescent microscope (Thermo Fisher Scientific). Red and green fluorescence intensity of each image was measured using ImageJ v.1.48, and the red/green ratio was calculated.

#### JC-1 staining of MSCs cultured in buffered hypercapnia

In some experiments, acidosis was buffered to facilitate the determination of the individual contributions of pH and CO_2_ to the effects of HCA, particularly its effects on mitochondrial membrane potential of MSCs. The experimental setup was done as in previous experiments, with both unstimulated or cytomix-stimulated MSCs being cultured in 5 or 15% CO_2_ for 24 h. However, in this case, an additional 2 groups (1 unstimulated and 1 cytomix-stimulated) in which the pH was buffered to that observed in 5% CO_2_ were included in 15% CO_2_. This was achieved using pre-equilibrated medium that contained 0.02 M sodium bicarbonate (NaHCO_3_) (MilliporeSigma). A total of 0.02 M sodium chloride (NaCl) (MilliporeSigma) prepared in pre-equilibrated medium was added to all other groups in both 5 and 15% CO_2_ to ensure all groups were equiosmolar. Cells were then cultured in 5 or 15% CO_2_ for 24 h prior to JC-1 staining as previously described in the cell culture section.

#### ATP assay

The majority of cellular ATP originates from the mitochondrial electron transport chain. ATP production is therefore indicative of mitochondrial function. Intracellular ATP levels were measured after culture of MSCs in the presence or absence of 50 ng/ml cytomix for 24 h using a luminescent ATP detection assay kit (Abcam, Cambridge, United Kingdom), following the manufacturer’s instructions. All reagents were brought to room temperature prior to use. Fifty microliters supplied detergent was added to each well containing the conditions of interest and to each well containing only 90 µl α-MEM_1% + PS + LG_ in preparation for a standard curve. The plate was incubated at room temperature on an orbital shaker for 5 min. Standards ranging in concentration from 10 µM to 0.1 nM were prepared. Ten microliters prepared standard was added to 90 µl α-MEM_1% + PS + LG_ already in the wells reserved for the standard curve. The plate was incubated at room temperature for 5 min on an orbital shaker. Fifty microliters of the supplied substrate solution was then added per well. The plate was incubated again at room temperature for 5 min on an orbital shaker. Luminescence was measured immediately using a Fluostar Omega microplate reader.

### Coculture experiments

#### Mitochondrial transfer

MitoTracker dyes (Thermo Fisher Scientific) are cell-permeable probes that react with the thiol groups of, and thus label, mitochondria. To investigate whether mitochondria are transferred from MSCs to SAECs, MSCs were stained with 125 ng/ml MitoTracker Green FM (Thermo Fisher Scientific) for 45 min at 37°C in 5% CO_2_. SAECs were prestained with 125 ng/ml MitoTracker Deep Red (Thermo Fisher Scientific) for 1 h at 37°C in 5% CO_2_. Stained cells were washed 3 times with 1× PBS. A sample of the third MSC wash was retained for use as a leak control. Prestained MSCs were seeded on Transwell inserts with 0.4-µm pores (Greiner Bio-One) at a ratio of 1 MSC to every 5 SAECs and cocultured for 24 h in 5 or 15% CO_2_ in the presence of 50 ng/ml cytomix with prestained SAECs seeded on the well surface below. For the leak control, MSCs were replaced with an equal volume of the MSC wash retained earlier.

After 24 h, the Transwells were removed and the SAECs were washed 3 times with PBS. Live SAECs were immediately imaged at ×20 magnification on an EVOS FL Auto epifluorescence microscope to visualize MSC-derived green mitochondria uptake by SAECs.

#### Coculture *in vitro* scratch assay

To assess the effects of MSCs on SAEC wound closure in normocapnia and HCA, MSCs were cocultured on Transwell inserts (Greiner Bio-One) with SAECs as previously described in the coculture experiments section. SAECs were wounded in an *in vitro* scratch assay. Plates were incubated at 37°C in 5 or 15% CO_2_ for 24 h. At 24 h, Transwell inserts were removed, and the wound areas above and below the horizontal lines were reimaged. The area of the wound site at baseline and 24 h was measured using ImageJ v.1.48. The values obtained were used to calculate the percentage wound closure over 24 h.

#### Ki67 staining

Ki67 is a cellular marker of proliferation. SAECs wounded in an *in vitro* scratch assay and cocultured with MSCs were immunofluorescently stained for this marker. SAECs were washed 3 times with 1× PBS and fixed with 4% paraformaldehyde at room temperature for 15 min. Excess paraformaldehyde was aspirated, and the wash step was repeated. Cells were permeabilized by incubation with 0.5% Triton X-100 for 20 min at room temperature. The wash step was repeated. Cells were blocked with 200 µl 1% BSA (prepared in 1× PBS) for 2 h at room temperature. The wash step was repeated. The primary antibody Ki67 mAb (SolA15) (Thermo Fisher Scientific) was diluted 1:200 in 1% BSA. One hundred microliters primary antibody was added per well and incubated at 4°C overnight. For a secondary-only control, 100 µl 1% BSA only was added.

After overnight incubation, cells were washed 3 times with 1× PBS. Goat anti-mouse/rat Alexa Fluor 594 was diluted 1:200 in 1% BSA. One hundred microliters was added per well and incubated in the dark for 1 h at room temperature. For a primary-only control, 100 µl 1% BSA only was used. The wash step was repeated. One hundred microliters Hoechst nuclear stain (undiluted) (MilliporeSigma) was added per well and incubated in the dark at room temperature for 20 min. The wash step was repeated. Two hundred microliters 1× PBS was added to the wells and the plates were stored at 4°C in the dark until imaging.

Images were taken at ×5 magnification using a Leica DMi8 microscope (Leica Microsystems, ‎Wetzlar‎, Germany). One image was taken above and 1 below the horizontal line running across the undersurface of the wells. To quantify Ki67 staining, a grid of individual squares each of 100,000 pixels^2^ in size was drawn over each image using ImageJ v.1.48. Four wells at the edge of the wound were selected from each image, and the number of cells stained positively for Ki67 were counted using the multipoint tool. The average number of Ki67-positive cells per 100,000 pixels^2^ was determined.

#### Loss-of-function experiment

A loss-of-function experiment was performed to determine the contribution of mitochondria to the ability of MSCs to promote SAEC wound closure in normocapnia. To induce mitochondrial dysfunction, confluent MSCs were treated with 1 µg/ml rhodamine 6G (R6G) (MilliporeSigma) prepared in α-MEM_16.5% + PS + LG_ for 48 h at 37°C in 5% CO_2_. Medium was supplemented with 50 µg/ml uridine (MilliporeSigma) and 2.5 mM sodium pyruvate (MilliporeSigma) to support glycolysis. At the same time, the medium in another flask of MSCs at the same passage and confluence was replaced with fresh α-MEM_16.5% + PS + LG_. After 48 h, the media were aspirated from both flasks of MSCs, and the cells were washed 3 times with PBS. The MSCs were trypsinized and counted. The *in vitro* scratch assay was repeated, including an additional group in normocapnia in which R6G-treated MSCs were cocultured with SAECs.

### Statistical analysis

GraphPad Prism v.5.01 (GraphPad Software, La Jolla, CA, USA) was used to perform statistical analysis. One-way ANOVA with Bonferroni *post hoc* analysis was used to analyze parametric data. Kruskal-Wallis with Dunn’s *post hoc* analysis was used for nonparametric data. Grouped data were analyzed by 2-way ANOVA. Values of *P* < 0.05 were regarded as statistically significant.

## RESULTS

### HCA attenuates the inflammatory response of HPMECs and SAECs

**Figure 1:**
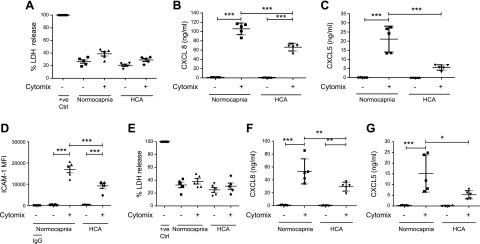
HCA attenuates the inflammatory response of HPMECs and SAECs. *A*) HCA did not alter HPMEC cell viability at 72 h when assessed by measurement of LDH release. *B*) HCA attenuated HPMEC secretion of the neutrophil chemoattractant CXCL8 at 72 h when measured by ELISA. *C*) HCA attenuated HPMEC secretion of the neutrophil chemoattractant CXCL5 at 72 h when measured by ELISA. *D*) HCA reduced HPMEC expression of the neutrophil adhesion molecule ICAM-1 at 72 h. This was reflected in the MFI when analyzed by flow cytometry. *E*) HCA did not alter SAEC viability at 24 h. *F*) HCA attenuated SAEC secretion of CXCL8 at 24 h. *G*) HCA attenuated SAEC secretion of CXCL5 at 24 h. +ve Ctrl, positive control. Scatter plots show means and sds. *n* = 5 per group for all experiments. *P < 0.05, **P < 0.01, ***P < 0.001.

To mimic the inflammatory environment of ARDS, endothelial and epithelial cells were stimulated with a pro-inflammatory cytokine mixture (IL-1β, INF-γ, and TNF-α) and cultured in either 5% CO_2_ (normocapnia) or 15% CO_2_ (HCA). The effects of HCA on both cell types were similar. HCA did not affect the viability of HPMECs for at least 72 h (**Fig. 1*A***). At 72 h, HCA significantly attenuated levels of HPMEC secretion of the neutrophil chemoattractants CXCL8 and CXCL5 (Fig. 1*B, C*) and the level of expression of neutrophil adhesion molecule ICAM-1 [as measured by median fluorescence intensity (MFI)] (Fig. 1*D*). No differences were observed in the percentage of cells expressing neutrophil adhesion molecules E-selectin or VCAM-1 or in the degree of expression per cell (Supplemental Fig. S1). As with HPMECs, viability of SAECs remained unaltered in HCA up to 72 h (Fig. 1*E*), whereas levels of CXCL8 and CXCL5 secretion were significantly decreased at 24 h (Fig. 1*F, G*).

### HCA attenuates wound repair by HPMECs and SAECs

**Figure 2:**
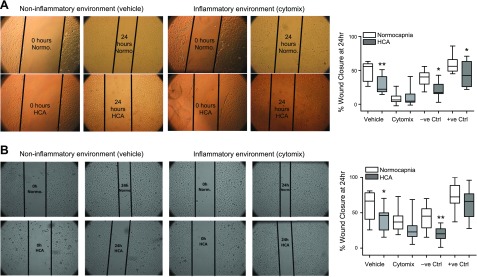
HCA attenuates wound repair by HPMECs and SAECs. Monolayers of HPMECs and SAECs were wounded in an *in vitro* scratch assay and exposed to normocapnia or HCA for 24 h. *A*) HCA significantly reduced percentage wound closure by HPMECs at 24 h in a noninflammatory (vehicle) but not inflammatory (cytomix) environment. These findings are corroborated by representative images depicting approximate wound areas at 0 and 24 h (*n* = 4 per group). *B*) HCA significantly reduced percentage wound closure by SAECs at 24 h in a noninflammatory (vehicle) but not inflammatory (cytomix) environment. These findings are corroborated by representative images depicting approximate wound areas at 0 and 24 h (*n* = 5 per group). Images were taken by Zeiss Axiovert 25 CFL microscope. +ve Ctrl, positive control. Box and whisker plots show median, first and third quartiles, and maximum and minimum values. Original magnification, ×20. *P < 0.05, **P < 0.01.

*In vitro* wound scratch assays revealed that HCA significantly impairs HPMEC and SAEC wound closure at 24 h in a noninflammatory setting (**Fig. 2**). When stimulated with cytomix, HPMEC wound closure was almost completely inhibited under both conditions (Fig. 2*A*). Similarly, SAEC wound closure was also diminished by cytomix stimulation in normocapnia, whereas a nonsignificant trend toward further worsening of the reparative response was observed in HCA (Fig. 2*B*).

### HCA does not alter the degree of NF-κB activation but impairs mitochondrial function

**Figure 3:**
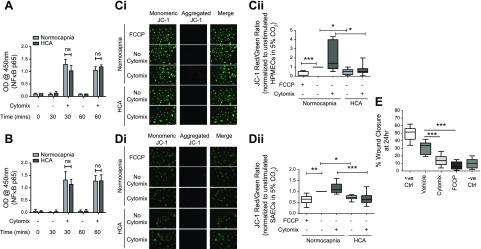
HCA does not alter the degree of cytomix-induced NF-κB activation but impairs mitochondrial function. *A*) When assessed based on the relative quantity of active p65 subunits present in nuclear extracts from HPMECs, HCA did not affect the degree of cytomix-induced NF-κB activation at 30 or 60 min (*n* = 3 per group). *B*) The degree of cytomix-induced NF-κB activation in SAECs was also unaffected by HCA at 30 and 60 min when assessed in the same way (*n* = 5 per group). *Ci*, *ii*) Staining with the mitochondrial membrane potential indicator JC-1 revealed significant attenuation of HPMEC mitochondrial membrane potential by HCA in both the presence and absence of cytomix stimulation at 24 h (*n* = 5 per group). *Ci*) Representative images. *Cii*) The JC-1 red (aggregate)/green (monomer) ratio was quantified in ImageJ and presented relative to unstimulated cells in normocapnia. *Di*, *ii*) Mitochondrial membrane potential in SAECs was also attenuated by HCA at 24 h (*n* = 4 per group). *Di*) Representative images. *Dii*) The JC-1 red (aggregate)/green (monomer) ratio was quantified in ImageJ and presented relative to unstimulated cells in normocapnia. *E*) Wound repair by HPMECs at 24 h was impaired by pharmacological dissipation of mitochondrial membrane potential using the mitochondrial uncoupler FCCP (*n* = 3 per group). Images were taken on an EVOS FL Auto epifluorescence microscope (*n* = 5 per group). Ctrl, control; ns, not significant; OD, optical density; +ve, positive control. Error bars (*A*, *B*) represent sd, and box and whisker plots show median, first and third quartiles, and maximum and minimum values. Original magnification, ×20. A value of *P* > 0.05 was not significant, *P < 0.05, **P < 0.01, ***P < 0.001.

It has been previously demonstrated that HCA attenuates the inflammatory responses of A549 cells through inhibition of NF-κB activation (14). To probe the mechanism behind the observed effects of HCA on HPMECs and SAECs, the degree of NF-κB activation was assessed by p65 TransAM assay, a DNA-binding ELISA by which consensus oligonucleotides are used to detect the presence of active NF-κB p65 subunits in nuclear extracts. Interestingly, in both cell types, the degree of NF-κB activation was not altered by HCA (**Fig. 3*A, B***).

Mitochondrial dysfunction has been reported as an alternative mechanism involved in the HCA effects. Specifically, it was associated with impaired proliferation of A549 cells (42). The effect of HCA on mitochondrial membrane potential was therefore investigated. Staining of both cell types with the mitochondrial membrane potential indicator JC-1 revealed significant attenuation of mitochondrial membrane potential—indicative of impaired mitochondrial function—under both inflammatory and noninflammatory conditions in HCA (Fig. 3*Ci*–*Dii*).

To further investigate the impact of reduced mitochondrial membrane potential on the reparative properties of HPMECs, mitochondrial membrane potential was pharmacologically dissipated using the mitochondrial uncoupler FCCP. Interestingly, FCCP treatment significantly abrogated HPMEC wound closure (Fig. 3*E*).

### HCA induces mitochondrial dysfunction in MSCs

**Figure 4:**
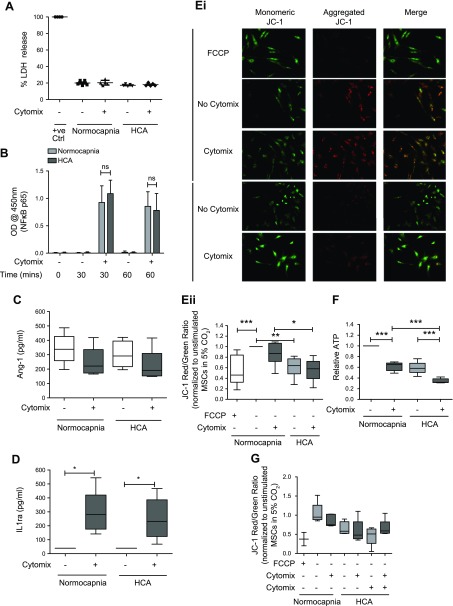
HCA induces mitochondrial dysfunction in MSCs. *A*) Viability of MSCs was unaffected by HCA at 24 h when assessed by measurement of LDH release (*n* = 2 per group). *B*) The degree of cytomix-induced NF-κB activation in MSCs was unaffected by HCA at 30 or 60 min when assessed based on the relative amount of active p65 subunits present in nuclear extracts obtained from MSCs (*n* = 5 per group for 0 and 60 min; *n* = 4 per group for 30 min). *C*) MSC secretion of the soluble mediator Ang-1 was not altered by HCA at 24 h when quantified by ELISA (*n* = 5 per group). *D*) The quantity of the soluble mediator IL-1ra secreted by MSCs was also unaffected by HCA at 24 h (*n* = 5 per group). *Ei*, *ii*) Staining with the mitochondrial membrane potential indicator JC-1 revealed significant attenuation of HPMEC mitochondrial membrane potential by HCA in both the presence and absence of cytomix stimulation at 24 h. *Ei*) Representative images. *Eii*) The JC-1 red (aggregate)/green (monomer) ratio was quantified in ImageJ and presented relative to unstimulated cells in normocapnia (*n* = 5 per group). *F*) HCA attenuated ATP production by MSCs at 24 h (*n* = 5 per group). *G*) Buffering medium pH to that of normocapnia using 0.02 M NaHCO_3_ did not alter the effect of HCA on mitochondrial membrane potential in MSCs at 24 h (*n* = 3 per group except FCCP and all groups not stimulated with cytomix in HCA, which are *n* = 2). Images were taken on an EVOS FL Auto epifluorescence microscope. Ctrl. Control; ns, not significant; OD, optical density; +ve, positive control. Scatter plots show mean and sd, and box and whisker plots show median, first and third quartiles, and maximum and minimum values. Original magnification, ×20. *P < 0.05, **P < 0.01, ***P < 0.001.

Next, the effects of HCA on human bone marrow–derived MSCs were tested. Similar to the observations in endothelial and epithelial cells, HCA did not alter the viability of MSCs (**Fig. 4*A***). The degree of NF-κB activation (Fig. 4*B*) and secretion of Ang-1 and IL-1ra (Fig. 4*C, D*) were also unaffected by HCA. Of note, both Ang-1 and IL-1ra are paracrine soluble factors known to be involved in mediating the therapeutic effects of MSCs in preclinical models of inflammatory lung disease (32, 43). The promotors of both genes are also under NF-κB regulation (44, 45), further supporting the finding that the effects of HCA on MSCs occur independently of alterations to the NF-κB pathway. Levels of keratinocyte growth factor and IL-10 secreted by MSCs were also analyzed, but concentrations were below the lower limits of detection of the assays (<31.25 pg/ml for both analytes, unpublished results). Again, analogous to its effect on HPMECs and SAECs, HCA impaired MSC mitochondrial function. This was evidenced by attenuated mitochondrial membrane potential (Fig. 4*Ei*, *ii*) and reduced ATP production by the MSCs (Fig. 4*F*). To buffer the medium pH to that of normocapnia, 0.02 M NaHCO_3_ was added to the medium under hypercapnic conditions. This did not alter the effect of HCA on MSC mitochondrial membrane potential (Fig. 4*G*).

### HCA-induced mitochondrial dysfunction impairs the capacity of MSCs to improve epithelial wound closure

**Figure 5:**
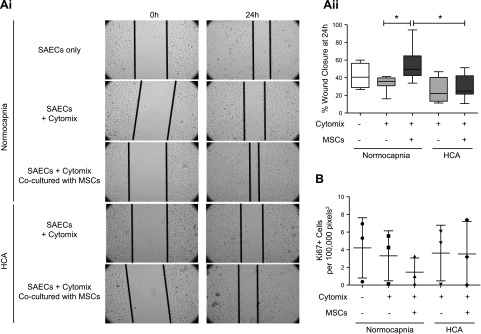
MSCs promote SAEC wound repair in normocapnia but not HCA. *Ai*, *ii*) MSCs in a noncontact (Transwell) coculture with SAECs that had been wounded in an *in vitro* scratch assay promoted SAEC wound closure in normocapnia. This effect was not observed in HCA at 24 h (*n* = 4 per group). *B*) Wounded SAECs that had been cultured with MSCs in a noncontact coculture were immunofluorescently stained for the proliferation marker Ki67. Imaging revealed no difference in proliferation across any of the conditions investigated (*n* = 3 per group). Images were taken by Zeiss Axiovert 25 CFL microscope *Ai*) Representative images. *Aii*) Percentage of wound closure over 24 hours was quantified for each condition. *Aii, B*) Box and whisker plots (*Aii*) show median, first and third quartiles, and maximum and minimum values; scatter plots (*B*) show mean and sd. Original magnification, ×20. *P < 0.05.

Next, the effect of HCA on the ability of MSCs to improve SAEC wound closure was investigated. In an *in vitro* wound scratch assay, the degree of SAEC wound closure was significantly enhanced by a noncontact coculture (Transwell) with MSCs in normocapnia. This effect was attenuated by HCA (**Fig. 5*Ai*, *ii***). Staining of the same SAECs for the proliferation marker Ki67 revealed that the degree of SAEC proliferation was not altered by MSCs (Fig. 5*B*).

**Figure 6:**
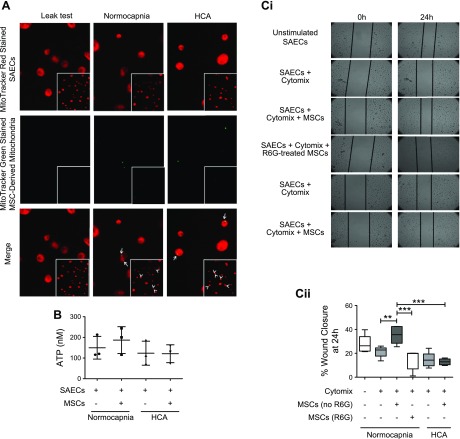
Impaired mitochondrial transfer mediates the loss of MSC therapeutic potential. *A*) Mitochondria within MSCs were stained with MitoTracker Green, whereas mitochondria within SAECs were labeled with MitoTracker Red. Stained MSCs were cultured in a noncontact (Transwell) coculture with stained SAECs. Mitochondrial transfer from MSCs to SAECs was observed by fluorescence microscopy at 24 h. Representative images. *B*) Coculture was repeated using unstained cells. MSCs induced a small, albeit insignificant, increase in ATP production at 24 h under conditions of normocapnia but not HCA (*n* = 3 per group). *Ci*, *ii*) MSCs were pretreated with R6G to induce mitochondrial dysfunction. These MSCs were cultured in a noncontact coculture with SAECs that had been wounded in an *in vitro* scratch assay. Untreated but not R6G-treated MSCs enhanced SAEC wound closure at 24 h in normocapnia. *Ci*) Representative images. *Cii*) Percentage of wound closure over 24 hours was quantified for each condition (*n* = 5–6 per group). Fluorescent images were taken on an EVOS FL Auto epifluorescence microscope, epithelial wounds (*Ci*) were imaged by Zeiss Axiovert 25 CFL microscope. Scatter plots (*B*) show mean and sd, and box and whisker plots (*Cii*) show median, first and third quartiles, and maximum and minimum values. Original magnification, ×20. **P < 0.01, ***P < 0.001.

Given that MSC secretion of soluble mediators was not affected by HCA, it was hypothesized that HCA-induced mitochondrial dysfunction could play a role in the loss of the MSC effect on epithelial repair. Previous studies demonstrated that transfer of mitochondria within extracellular vesicles is important for the therapeutic mechanism of MSCs in LPS-induced lung injury (36, 46). To test this hypothesis, the ability to detect MSC mitochondria within SAECs after incubation with MSC conditioned medium (CM) was investigated. MSCs were prestained with MitoTracker Green. Their CM was collected and added to SAECs, which had been prestained with MitoTracker Red FM. Fluorescence microscopy after 24-h culture of SAECs with CM revealed evidence of colocalization of MSC-derived mitochondria with endogenous mitochondria of SAECs (**Fig. 6*A***, yellow staining, white arrows). No difference in the extent of mitochondrial transfer between normocapnic and hypercapnic conditions was observed.

Consistent with the above observations demonstrating a detrimental effect of HCA on mitochondrial membrane potential across all 3 cell types studied, HCA significantly reduced ATP production by SAECs (Fig. 6*B*). Additionally, whereas MSC-CM enhanced ATP production by SAECs in normocapnia (albeit nonsignificantly), ATP levels were not restored by MSC-CM in the setting of HCA (Fig. 6*B*).

To further investigate the requirement of functional mitochondria for the ability of MSCs to promote SAEC wound repair, the *in vitro* scratch assay was repeated using MSCs in which mitochondrial dysfunction had been induced by pretreatment with R6G. R6G irreversibly impairs oxidative phosphorylation but does not significantly impair MSC secretion of soluble mediators (36). Although MSCs promoted SAEC wound closure in normocapnia, this effect was lost with R6G-pretreated MSCs (Fig. 6*Ci*, *ii*).

## DISCUSSION

Low tidal volume ventilation is the mainstay of treatment in ARDS, but it can be associated with the development of HCA (2). The capillary endothelium and alveolar epithelium are key targets in the pathogenesis of ARDS; restoration of their structure and function is required for resolution of the condition. To date, the effects of HCA on the pulmonary capillary endothelium are unclear, although many investigations into its effects on the alveolar epithelium have been limited to cell lines such as A549. The current study demonstrates the effects of HCA on primary HPMECs and the SAECs, using a degree of hypercapnia equivalent to pCO_2_ 9.0 kPa (68 mmHg). This degree of hypercapnia is believed to be clinically relevant, with a recent observational study having demonstrated that 13% of patients with ARDS develop severe hypercapnia of >60 mmHg.

The main findings from this study are as follows: *1*) HCA attenuates inflammatory responses in primary HPMECs and distal lung epithelial cells while it simultaneously impairs their reparative capacities (Figs. 1 and 2); *2*) HCA induces mitochondrial dysfunction in primary human pulmonary endothelial and epithelial cells as well as bone marrow–derived MSCs (Figs. 3 and 4); and *3*), whereas MSCs improve epithelial wound closure through transfer of functional mitochondria within extracellular vesicles, HCA-induced mitochondrial dysfunction diminishes the therapeutic effect of MSCs on epithelial wound repair in the inflammatory environment (Figs. 5 and 6).

Neutrophils are key mediators of inflammation and, subsequently, tissue damage in ARDS. Their alveolar recruitment depends in part on chemotactic stimuli such as CXCL5 and CXCL8 (47). In the present study, secretion of these chemokines by both HPMECs and SAECs was attenuated in HCA (Fig. 1). These data are in accordance with previous *in vitro* observations using macrovascular endothelial cells (4) and A549 cells (14), but this is the first report of the effects of HCA on CXCL5 secretion by HPMECs and SAECs.

Endothelial expression of neutrophil adhesion molecules also contributes to alveolar neutrophil recruitment. Although there were no differences in the percentage of cells expressing adhesion molecules ICAM-1, VCAM-1, or E-selectin, the degree of surface expression of ICAM-1 (reflected in the MFI) was significantly attenuated in HCA. This finding is again in keeping with previous observations in macrovascular endothelial cells (4).

Attenuated neutrophilia in HCA has been demonstrated in numerous *in vivo* studies (11, 12, 48). Data from the current study suggest that the epithelium and endothelium contribute to this response by attenuating neutrophil chemoattraction and migration across the endothelial-epithelial barrier.

Integrity of the epithelial and endothelial barriers are key to regulating neutrophil recruitment, particularly in ARDS, where basement membranes are often denuded. Following cell activation, cytoskeletal remodeling occurs, promoting cell migration in an attempt to restore barrier integrity. *In vitro* wound scratch assays revealed that HCA significantly impaired wound repair in a noninflammatory environment and demonstrated a trend toward further worsening in an inflammatory setting (Fig. 2). These findings contrast with a previous report in which endothelial wound repair was not altered by HCA (49), although this discrepancy may be explained by the use of macrovascular endothelial cells. The findings in SAECs, however, corroborate and extend previous observations by O’Toole *et al.* (15).

Surprisingly, although altered NF-κB activation has consistently been attributed to the effects of HCA (14, 15), the results of the current study suggest that HCA-induced alterations in inflammatory responses and wound repair occurred independently of NF-κB. To date, impaired mitochondrial function is the only alternative mechanism shown to mediate HCA-driven effects (42). In the current study, HCA induces significant attenuation of mitochondrial membrane potential in all 3 primary cell types studied, indicating mitochondrial dysfunction (Figs. 3 and 4). Previous work has linked alterations in mitochondrial membrane potential with the development of an anti-inflammatory phenotype in macrophages (50). In addition, low mitochondrial membrane potential has been associated with the induction of mitophagy (51, 52). Mitophagy has been reported to confer resistance to cell death in both alveolar epithelial cells and macrophages (53, 54), suggesting that HCA may be inducing mitophagy to maintain cell viability in this study. Low mitochondrial membrane potential and mitophagy have also been previously associated with impaired proliferation and migration (55–57), 2 factors contributing to dysregulated tissue repair in ARDS. In the present study, pharmacological inhibition of mitochondrial membrane potential resulted in significant abrogation of HPMEC wound closure (Fig. 3*E*), suggesting that functional mitochondria are important for endothelial repair. Thus, HCA-induced mitochondrial dysfunction could negatively affect the reparative capacity of the injured lung in the clinical setting.

From a therapeutic perspective, although MSCs are a promising candidate for ARDS (58), their efficacy has never been studied in the setting of HCA. It is well established that MSCs secrete a plethora of soluble factors, including Ang-1 and IL-1ra, which are thought to mediate, at least in part, the therapeutic effects of MSCs (34). The current study demonstrates that MSC secretion of these NF-κB–regulated molecules was unaltered by HCA. The degree of cytomix-induced NF-κB activation was also unaffected, suggesting that in HCA MSCs retain their capacity to exert therapeutic effects mediated by these soluble factors (Fig. 4*B–D*). Nevertheless, the therapeutic effect of MSCs on pulmonary epithelial wound repair was abrogated in HCA [despite MSCs promoting SAEC wound closure *via* enhanced migration in normocapnia, as previously reported in Akram *et al.* (59) (Fig. 5). The lack of therapeutic effect of MSCs may be attributed to impaired mitochondrial function in the presence of HCA. This was reflected by attenuation of both mitochondrial membrane potential and intracellular ATP production (Fig. 4*Ei*–*F*). Importantly, buffering of acidosis did not alter the effects of HCA on MSC mitochondrial membrane potential (Fig. 4*G*), suggesting that this effect occurs independently of pH. This HCA-induced mitochondrial damage in MSCs suggests that MSCs may lose their capacity to improve epithelial repair in patients with ARDS who develop HCA.

Mitochondrial transfer to surrounding injured cells has previously been shown to be important for the therapeutic potential of MSCs (35, 36, 53, 54). Here, mitochondrial transfer from MSCs to SAECs was observed in both normocapnia and HCA and was associated with enhanced ATP production by SAECs in normocapnia (Fig. 6*A, B*). To investigate whether mitochondrial transfer was responsible for the MSC-mediated improvement in SAEC wound closure in normocapnia, the *in vitro* wound scratch assay was repeated using MSCs with dysfunctional mitochondria. To achieve this, MSCs had been pretreated with R6G, which induces mitochondrial dysfunction without significantly affecting other aspects of cell biology, such as the ability to secrete soluble molecules (36, 60). The results demonstrate, for the first time, that functional mitochondria are required for the ability of MSCs to promote primary human distal lung epithelial repair (Fig. 6*Ci*, *ii*). This reveals a new mechanism of the therapeutic effects of MSCs in ARDS and implies that HCA-induced mitochondrial dysfunction was responsible for loss of the MSC effect in this model. Given that transfer of functional mitochondria was previously shown to be responsible for MSC modulation of both the phenotype of alveolar macrophages (35, 36) and of surfactant secretion by alveolar epithelial type II cells (46) in preclinical models of ARDS, this finding raises significant concerns regarding the therapeutic efficacy of MSCs in patients with HCA.

Although this study provides novel results in relation to HCA-mediated mitochondrial damage in human pulmonary epithelial and endothelial cells as well as MSCs, further investigations into the mechanisms of this effect are required. Also, although a role for mitochondrial dysfunction in HCA-induced inhibition of wound repair was demonstrated, the mechanism responsible for the anti-inflammatory effect observed in primary cells in HCA remains unclear. Finally, use of a broader range of clinically relevant models utilizing human lung tissues (*e.g.*, *ex vivo* perfused lungs, lung-on-a-chip, or precision-cut lung slices) will facilitate better understanding of the impact of HCA on human lung repair.

Despite HCA exerting an anti-inflammatory effect, it also appears to attenuate the reparative capacity of pulmonary endothelial and epithelial cells and reduce the therapeutic efficacy of MSCs in ARDS. This suggests that when interpreting clinical trial data, *a priori* *post hoc* analysis of data from patients with and without HCA may be useful, as any protective benefit of an MSC-based therapy may be masked by the lack of an effect in patients with HCA. This may subsequently allow for appropriate stratification of patients with ARDS who are most likely to respond to MSC-based therapies.

There are undoubtedly a number of limitations to the work presented. For example, whereas a previous report (61) suggested that the effects of hypercapnia on NF-κB activation are rapidly reversible, the current study does not address the potential reversibility of the effects of HCA on the anti-inflammatory and reparative capacities of HPMECs and SAECs or on the therapeutic potential of MSCs in ARDS. If the effects of HCA are found to be reversible, it is perceivable that extracorporeal CO_2_ removal could potentially manage the effects in patients with ARDS. However, although this may benefit the impaired therapeutic capacity of MSCs, which appears to result from HCA, it will undoubtedly be difficult to achieve a perfect balance between the effects of HCA on the inflammatory and reparative capacities of HPMECs and SAECs.

In summary, HCA-induced mitochondrial dysfunction may contribute to impaired epithelial and endothelial repair, resulting in worse outcomes for patients with severe lung disease. Additionally, patients with HCA may not be responsive to treatment with MSCs.

## Supplementary Material

This article includes supplemental data. Please visit *http://www.fasebj.org* to obtain this information.

## Abbreviations

α-MEM: minimum essential medium with alpha modifications
Ang-1: angiopoietin-1
ARDS: acute respiratory distress syndrome
BSA: bovine serum albumin
CM: conditioned medium
CXCL: C-X-C motif chemokine ligand
DPBS: Dulbecco’s phosphate-buffered saline
E-selectin: endothelial-selectin
FBS: fetal bovine serum
FCCP: carbonyl cyanide p-trifluoromethoxyphenylhydrazone
HCA: hypercapnic acidosis
HPMEC: human pulmonary microvascular endothelial cell
ICAM: intercellular adhesion molecule
IL-1ra: IL-1 receptor antagonist
JC-1: 5,5′,6,6′-tetrachloro-1,1′,3,3′-tetraethylbenzimidazolylcarbocyanine iodide
LDH: lactate dehydrogenase
LG: L-glutamine
MFI: median fluorescence intensity
MSC: mesenchymal stem cell
pCO_2_: partial pressure of CO_2_
PS: penicillin-streptomycin
R6G: rhodamine 6G
SAEC: small airway epithelial cell
