# Supplementary material for: Hypercapnic acidosis induces mitochondrial dysfunction and impairs the ability of mesenchymal stem cells to promote distal lung epithelial repair
Source: FASEB J. 2019 Jan 16;33(4):5585–98. doi: 10.1096/fj.201802056R (PMC6436662; doi:10.1096/fj.201802056R)
Supplement: Supplementary file 1 [file fj.201802056R.sf1.docx]

**Supplement 1**

**A**

**B**

**C**

**D**

**E**

**The effect of HCA on the expression of neutrophil adhesion molecules.** HPMECs cultured in 5% or 15% CO_2_ in the presence or absence of cytomix for 72 hours were harvested, immunostained and assessed by flow cytometry for expression of neutrophil adhesion molecules. **(A)** The percentage of HPMECs expressing ICAM-1 was unaffected by HCA. **(B)** The percentage of HPMECs expressing E-selectin was unaffected HCA. **(C)** The degree to which E-selectin was expressed per cell was unaffected by HCA, as reflected by the median fluorescence intensity (MFI). **(D)** The percentage of HPMECs expressing VCAM-1 was unaffected HCA. **(E)** The degree to which VCAM-1 was expressed per cell was unaffected by HCA, as reflected by the MFI. n=5 per group. Error bars represent standard deviation (SD). ns = p>0.05; *p<0.05; ***p<0.001.
